# Dupilumab Initiation for Surgically Refractory Chronic Rhinosinusitis With Nasal Polyps Is Associated With Differential Expression of CLC and Activation of Eicosanoid Pathways

**DOI:** 10.1002/alr.70019

**Published:** 2025-08-18

**Authors:** Christina Dorismond, Rory J. Lubner, Daniel H. Lofgren, Chandler J. Rygalski, Ping Li, Katherine N. Cahill, Suman R. Das, Mason R. Krysinski, Rakesh K. Chandra, Justin H. Turner, Naweed I. Chowdhury

**Affiliations:** ^1^ Department of Otolaryngology‐Head and Neck Surgery Vanderbilt University Medical Center Nashville Tennessee USA; ^2^ Division of Allergy, Pulmonary, and Critical Care Medicine, Department of Medicine Vanderbilt University Medical Center Nashville Tennessee USA; ^3^ Department of Pathology, Microbiology, and Immunology Vanderbilt University Medical Center Nashville Tennessee USA; ^4^ Department of Otolaryngology‐Head and Neck Surgery University of Mississippi Medical Center Jackson Mississippi USA; ^5^ Department of Otolaryngology‐Head and Neck Surgery University of Alabama‐Birmingham Birmingham Alabama USA

**Keywords:** chronic rhinosinusitis, differential gene expression, dupilumab, nasal polyp, transcriptome

## Abstract

- An untargeted transcriptomic analysis provided exploratory insight into key genes and pathways that can be used to help identify patients at risk for surgically refractory chronic rhinosinusitis with nasal polyps (CRSwNP).
- The Charcot–Leyden crystal gene, periostin and osteopontin‐associated pathways, eicosanoid synthesis pathway, and prostaglandin/leukotriene metabolism pathway, among others, are associated with dupilumab prescription for surgically refractory chronic rhinosinusitis and may support early stratification of disease severity in CRSwNP patients.

**Abbreviations**
AERD: aspirin‐exacerbated respiratory disease
CLC: Charcot–Leyden crystal
CRSwNP: chronic rhinosinusitis with nasal polyps
DEG: differentially expressed genes
ESS: endoscopic sinus surgery
GSEA: gene set enrichment analysis
IL: interleukin
LogFC: log fold‐change
NES: normalized enrichment score

## 1 Introduction

Monoclonal antibodies targeting type‐2 inflammation, such as dupilumab, are a recent addition to the treatment of chronic rhinosinusitis with nasal polyps (CRSwNP), often utilized in patients with disease recurrence following endoscopic sinus surgery (ESS). However, identifying patients at risk for recurrence preoperatively remains a challenge. We previously identified aspirin‐exacerbated respiratory disease (AERD), asthma, and mucus interleukin (IL)‐5 and IL‐13 levels as potential biomarkers associated with post‐ESS dupilumab prescription [1]. We hypothesized that an untargeted approach using transcriptomic data from nasal epithelial cells could provide additional insight into key pathways relevant to the identification of surgically refractory CRSwNP.

## 2 Methods

Patients undergoing ESS for CRSwNP were enrolled in a prospective study and followed for at least 6 months. Demographic variables, comorbid conditions, and dupilumab prescription status were obtained from patients’ medical records. At the beginning of surgery, nasal swabs were placed in the middle meatus to collect superficial nasal epithelial and inflammatory cells. RNA was then extracted and processed as previously described [2]. Detailed enrollment and laboratory methods are described in the Supporting Information.

Bulk transcriptional reads mapping to the human genome (hg38) were isolated and used for subsequent expression analysis using the *limma* package, with differentially expressed genes (DEGs) explored using volcano plots and cluster analysis. Afterwards, gene set enrichment analysis (GSEA) was conducted using *clusterProfiler* with the WikiPathways collection reference. Due to statistically significant differences in the sex distribution and AERD prevalence between groups (Table S1), both models were adjusted for these factors. We also adjusted the DEG analysis for tissue eosinophil counts, as this was nearly significantly different (*p* = 0.068). Detailed statistical methods are included in the Supporting Information.

## 3 Results

Of the 53 included patients, 20 (37.7%) were prescribed dupilumab during the study period. Demographic and clinical data are shown in Table S1. Notably, there were more women (60% vs. 30.3%, *p* = 0.033) and AERD patients (60% vs. 15.2%, *p* < 0.001) in the group prescribed dupilumab, with a similar prevalence of asthma, allergic rhinitis, prior surgery, and radiographic disease severity between groups.

**FIGURE 1:**
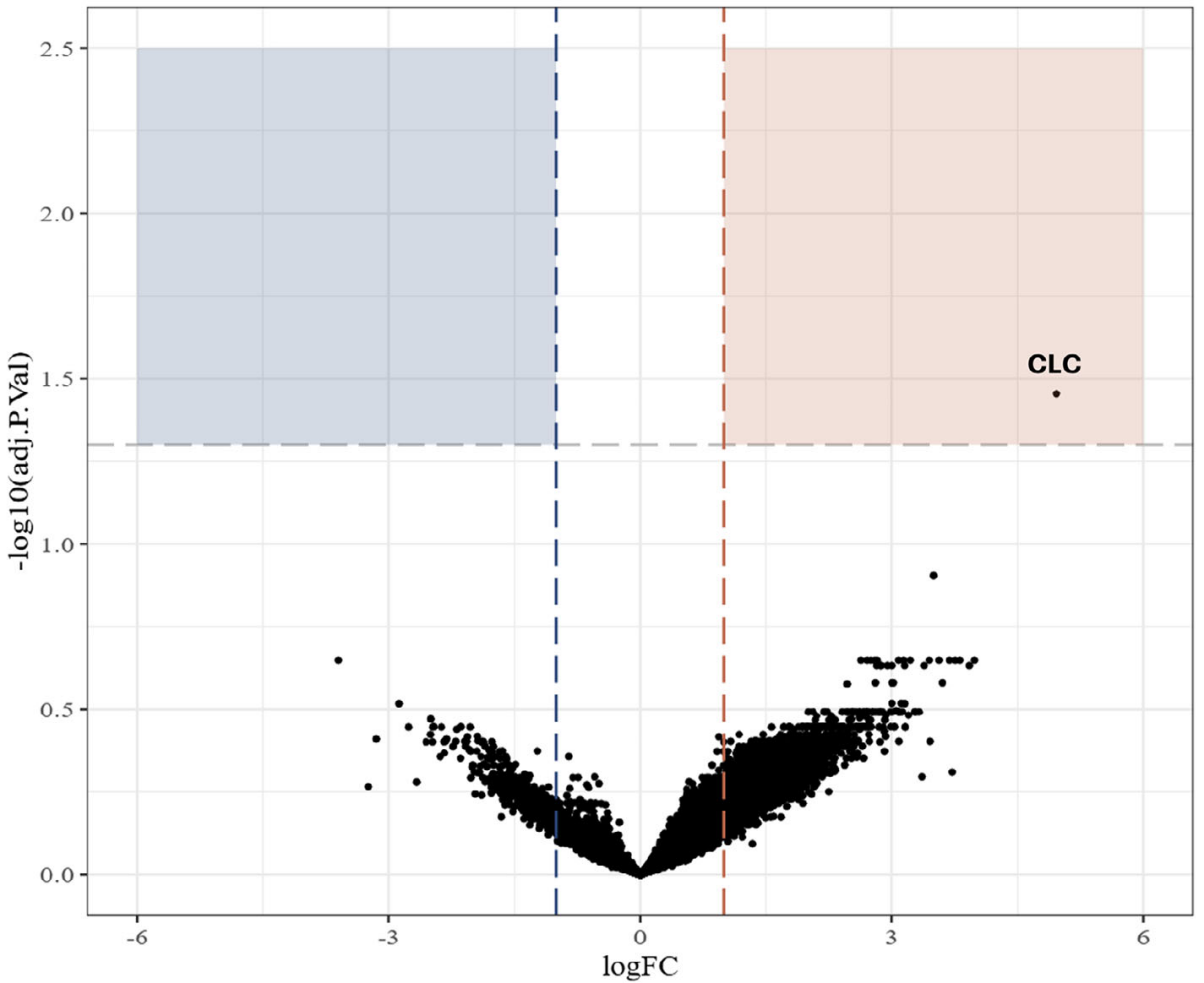
Volcano plot for differentially expressed genes based on postoperative dupilumab prescriptions, accounting for population differences in aspirin sensitivity, biologic sex, and tissue eosinophil counts. Genes to the right are more frequently expressed in participants who were prescribed dupilumab, while genes to the left are less frequently expressed. Only one gene (CLC, log FC = 4.96, adjusted *p*‐value = 0.035) associated with the galectin‐10 protein was statistically significant after accounting for multiple comparisons. CLC = Charcot–Leyden crystals; log FC = log fold‐change.

**FIGURE 2:**
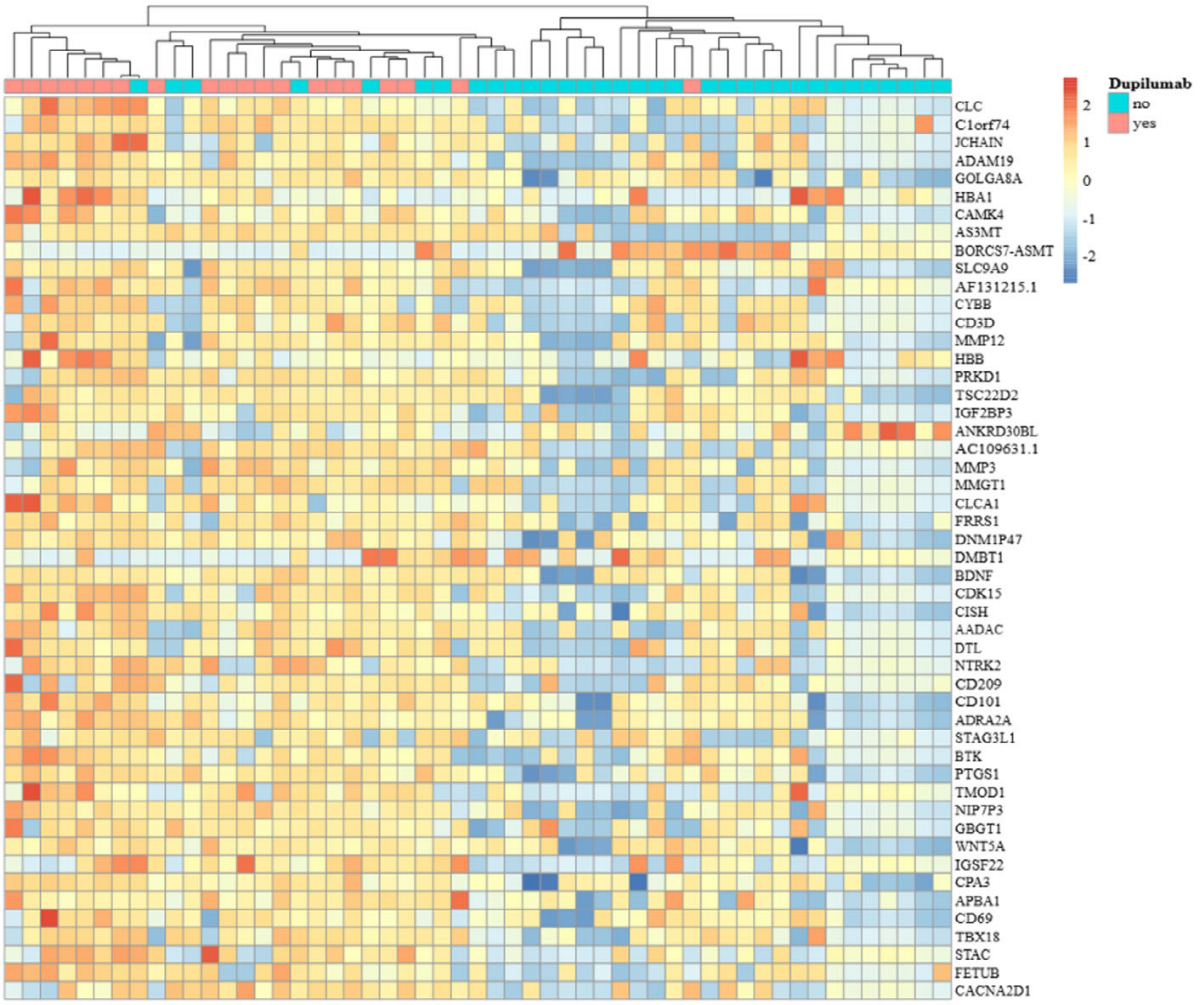
Heatmap for the top 50 differentially expressed genes based on postoperative dupilumab prescriptions, accounting for population differences in aspirin sensitivity, tissue eosinophil counts, and biologic sex. Rows ordered based on the absolute value of log fold‐change, with red colors signifying overexpression and blue colors signifying underexpression. Note clustering of most participants prescribed dupilumab on the left, with overexpression of the Charcot–Leyden crystal (CLC) protein compared to participants who were not.

On both adjusted (log fold‐change [logFC] = 4.96, FDR‐adjusted *p* [p.adj] = 0.035) and unadjusted (log FC = 4.63, adjusted *p* = 0.004) differential expression analysis, the Charcot–Leyden crystal (CLC) was the only gene significantly upregulated in patients prescribed dupilumab compared to those not prescribed dupilumab (Figure 1). We also performed cluster analysis with the top 50 DEGs ranked by log FC to identify other potential candidate genes associated with dupilumab prescription (Figure 2). It was found that the clustering approach grouped most dupilumab‐prescribed patients together based on these DEGs.

On unadjusted GSEA (Table S2), the top differentially enriched pathway in dupilumab‐treated patients was associated with periostin (POSTN, normalized enrichment score [NES] = 1.81, p.adj = 0.01). This periostin pathway was not enriched after accounting for AERD and sex, but the top pathway on adjusted analysis featured osteopontin (SPP1, NES = 1.78, p.adj < 0.01) (Table S3).

Pathways associated with eicosanoid synthesis and prostaglandin/leukotriene metabolism were also enriched in patients prescribed dupilumab on unadjusted GSEA (NES = 1.71, p.adj = 0.02 and NES = 1.71, p.adj = 0.01, respectively). The eicosanoid synthesis pathway remained enriched in the adjusted GSEA (NES = 1.71, p.adj = 0.04).

## 4 Discussion

In this exploratory study, we used an untargeted transcriptomic approach to identify genes and pathways that may serve as predictive biomarkers of surgically refractory CRSwNP. We found that the CLC gene, encoding galectin‐10, was upregulated in patients prescribed dupilumab. Galectin‐10 is a prominent protein in eosinophils that has been shown to promote type‐2 inflammation [3]. A previous report from China demonstrated that galectin‐10 mRNA expression levels correlate with recalcitrant CRSwNP, supporting our findings of its potential role as a predictive biomarker [4].

We also determined that the clustering analysis grouped most dupilumab‐prescribed patients together, suggesting that these candidate genes may be worth further investigation as predictive biomarkers (Figure 2). Given the increasingly recognized roles of mast cells [5] and M2 macrophages [6] in severe CRSwNP, transcriptional activity in the DEGs encoding mast cell carboxypeptidase A3 (CPA3), and macrophage‐derived extracellular matrix remodeling metallopeptidases such as ADAM19, MMP3, and MMP12 may be promising targets.

In the unadjusted GSEA, a periostin‐associated pathway was the most differentially enriched. Encoded by POSTN, periostin is a matricellular protein thought to lead to fibroblast differentiation and polyp formation in CRSwNP [7]. Periostin levels and POSTN mRNA expression have consistently been shown to be higher in patients with more severe CRSwNP, supporting our findings [7]. While this periostin pathway was not enriched after accounting for AERD and sex differences, the top adjusted pathway did feature osteopontin (SPP1), a known promoter of eosinophilic inflammation and extracellular matrix deposition with similar functions to periostin in airway disease [8].

The GSEA also featured eicosanoid synthesis and prostaglandin/leukotriene metabolism pathways. In CRSwNP and particularly in AERD, there is disruption of eicosanoid synthesis and metabolism, with a shift toward leukotrienes and pro‐inflammatory prostaglandins [9, 10]. The persistent enrichment of the eicosanoid synthesis pathway, even when adjusting for AERD, suggests a role for eicosanoids to stratify disease severity even in non‐AERD‐associated CRSwNP.

Together, these findings support the hypothesis that surgically refractory CRSwNP patients are characterized by high levels of eosinophilic infiltration, alterations in arachidonic acid metabolism, and overexpression of extracellular remodeling proteins. This combination of factors may lead to an inability of the sinonasal epithelium to reverse the aberrant epithelial–mesenchymal transformation seen in CRSwNP, perhaps through epigenetic alterations in basal cells [11].

Limitations include the study's exploratory nature, inability to account for subjective factors influencing dupilumab prescription, utilization of bulk‐RNA sequencing, and lack of confirmatory protein expression for the transcriptional findings. Nevertheless, this study provides a helpful basis for identifying potential predictors of surgically refractory CRSwNP and provides initial data to support the use of transcriptomics to stratify CRS patients for clinical purposes.

## Conflicts of Interest

Disclosure of potential conflicts of interest: R. K. Chandra: consultant to Sanofi, Regeneron, Optinose, Lyra Therapeutics. J. H. Turner has received grant support from the NIH/National Institute of Allergy and Infectious Diseases (NIAID) and NIH/National Institute on Aging (NIA) and personal fees from Regeneron. N. I. Chowdhury has received grant support from the Burroughs Wellcome Fund, the NIH/National Cancer Institute, NIH/National Institute of Allergy and Infectious Diseases (NIAID). C. Dorismond is a consultant for Myelin Healthcare. K. N. Cahill served on scientific advisory boards for AstraZeneca, Sanofi, Genentech, Regeneron, and Novartis, served as a consultant for Eli Lilly, reports royalties from UpToDate, and reports research support from Novo Nordisk. The remaining authors declare no conflicts of interest.

## Supporting information




**Supporting File 1**: alr70019‐supinfo‐0001
