# Supplementary material for: Dupilumab Initiation for Surgically Refractory Chronic Rhinosinusitis With Nasal Polyps Is Associated With Differential Expression of CLC and Activation of Eicosanoid Pathways
Source: Int Forum Allergy Rhinol. 2025 Aug 18;15(11):1296–9. doi: 10.1002/alr.70019 (PMC12581870; doi:10.1002/alr.70019)
Supplement: Supplementary file 1 — Supporting File 1: alr70019‐supinfo‐0001 [file ALR-15-1296-s001.docx]

**Dupilumab Initiation for Surgically Refractory Chronic Rhinosinusitis with Nasal Polyps is Associated with Differential Expression of CLC and Activation of Eicosanoid Pathways**

**Supplementary Text and Tables**

**Methods**

*Recruitment and Inclusion/Exclusion Criteria*

Adult patients presenting to Vanderbilt University Medical Center for ESS for a clinical diagnosis of chronic rhinosinusitis with nasal polyps were eligible for inclusion in this study based on EPOS^1^ or ICAR:RS^2^ criteria after failing a trial of appropriate medical management including topical nasal corticosteroids, at least one course of oral antibiotics and steroids, and nasal saline irrigations. Patients were excluded if they had CRS without nasal polyps, odontogenic rhinosinusitis, mycetoma, known autoimmune or granulomatous disorder, cystic fibrosis, recent systemic steroid use (within 4 weeks of surgery), or initiation of dupilumab prior to ESS. Tissue eosinophil counts were obtained from review of hematoxylin and eosin-stained histopathology slides by a board-certified pathologist, with the average count over 5 high power fields used as the representative value. Patients included in the study were selected as a convenience sample from a cohort of patients who met the inclusion and exclusion criteria and had transcriptomic data sequenced (n=84 CRS patients total, of which 53 had CRSwNP). This set of 84 patients was randomly selected from our overall CRS patient population enrolled in our ongoing prospective observational study. All sequenced CRSwNP patients had least 6 months of postoperative clinical follow-up, so no patients were excluded because of being lost to follow up. Dupilumab initiation data was collected after sequencing was completed and was not used as part of the selection process to determine which CRSwNP patients were included in the study.

*Post-Operative Course*

Therapy following ESS varied slightly between providers but generally included a course of systemic steroids, daily nasal corticosteroid sprays or steroid irrigations, and nasal saline rinses. The date dupilumab was prescribed for any indication was determined through review of medical records. Patients were included in the dupilumab group if they were prescribed dupilumab anytime during the study period and regardless of whether they were able to initiate dupilumab therapy.

*Cell Collection and Sequencing Methods*

At the beginning of surgery, a nasal swab was used to collect superficial cell samples from the middle meatus bilaterally under endoscopic guidance and placed into a tube containing 500μL of OMNIgene ORAL (DNA Genotek), which was frozen at -80°C and stored. Samples were later thawed and vortexed for 2 minutes, with 250µL of sample used for total RNA extraction. Briefly, the aliquot was homogenized in 600µL QIAzol (Qiagen) and 500µL of 2.0 mm zirconium oxide beads (Next Advance) using a Bullet Blender homogenizer, making sure to maintain temperatures at or near 4°C using the dry ice cooling system in the Bullet Blender. The sample was then treated with 100µL of genomic DNA Eliminator solution (Qiagen) to remove the genomic DNA and 180µL of chloroform was added to the samples for phase separation. The total RNA in the aqueous phase was then purified using the RNeasy Mini spin columns as recommended by the Qiagen RNeasy protocol. RNA integrity and RNA quantification were assessed using an Agilent Bioanalyzer RNA 6000 Nano/Pico Chip (Agilent Technologies, Palo Alto, California). Eukaryotic ribosomal RNAs (rRNA) were depleted using the NEBNext rRNA Depletion Kit, and after rRNA depletion, the samples were checked to ensure depletion of the 18S and 28S ribosomal peaks. Next, Illumina sequencing libraries were made using the NEBNext Ultra II RNA Library Prep Kit (NEB #E7775), with library quality assessment using an Agilent Bioanalyzer DNA High Sensitivity chip. Sequencing was then performed on an Illumina NovaSeq6000 platform with 2x150 base pair reads, with a sequencing depth of ~40 million paired-end reads per sample. Adapter removal and quality-based trimming of the raw reads were performed using *trimmomatic* using default parameters.^3^ Trimmed reads shorter than 50nt were discarded. FastQC was used before and after trimming to assess read quality.^4^

*Differential Expression Analysis and Statistical Methods*

The preprocessed reads were mapped to a reference transcriptome index generated from the hg38 genome and quantified using *kallisto*.^5^ Samples with fewer than 1 million pseudoaligned reads were filtered. The feature counts were combined into a single data frame using a custom R script. Differential expression analysis was then performed by comparing postoperative dupilumab prescription groups using the *limma* package.^6^ Genes with an absolute value log2 fold change > 1.5 with an adjusted p-value <0.05 were treated as differentially expressed. Gene set enrichment analysis (GSEA) for human sets in the WikiPathways reference database^7^ was performed using *clusterProfiler,^8^* with a false discovery rate of 0.05 used to determine statistical significance. Between group differences were compared using the Wilcoxon rank sum test for continuous variables or Pearson’s Chi-squared test for categorical variables, with an alpha level of 0.05 for statistical significance.

**References**

1. Fokkens WJ, Lund VJ, Hopkins C, et al. European Position Paper on Rhinosinusitis and Nasal Polyps 2020. *Rhinology*. Feb 20 2020;58(Suppl S29):1-464. doi:10.4193/Rhin20.600

2. Orlandi RR, Kingdom TT, Smith TL, et al. International consensus statement on allergy and rhinology: rhinosinusitis 2021. *Int Forum Allergy Rhinol*. Mar 2021;11(3):213-739. doi:10.1002/alr.22741

3. Bolger AM, Lohse M, Usadel B. Trimmomatic: a flexible trimmer for Illumina sequence data. *Bioinformatics*. Aug 1 2014;30(15):2114-20. doi:10.1093/bioinformatics/btu170

4. Andrews S. FastQC: a quality control tool for high throughput sequence data. 2010. <http://www.bioinformatics.babraham.ac.uk/projects/fastqc>

5. Bray NL, Pimentel H, Melsted P, Pachter L. Near-optimal probabilistic RNA-seq quantification. *Nat Biotechnol*. May 2016;34(5):525-7. doi:10.1038/nbt.3519

6. Ritchie ME, Phipson B, Wu D, et al. limma powers differential expression analyses for RNA-sequencing and microarray studies. *Nucleic Acids Res*. Apr 20 2015;43(7):e47. doi:10.1093/nar/gkv007

7. Agrawal A, Balci H, Hanspers K, et al. WikiPathways 2024: next generation pathway database. *Nucleic Acids Res*. Jan 5 2024;52(D1):D679-D689. doi:10.1093/nar/gkad960

8. Yu G, Wang LG, Han Y, He QY. clusterProfiler: an R package for comparing biological themes among gene clusters. *OMICS*. May 2012;16(5):284-7. doi:10.1089/omi.2011.0118

**Table S1.** Patient characteristics for all patients and by dupilumab prescription status.

| **Characteristic** | **Postoperative Dupilumab Initiation** | | **p-value***^2^* | | **Overall** | |  |
| --- | --- | --- | --- | --- | --- | --- | --- |
|  | **Yes**, N = 20*^1^* | **No**, N = 33*^1^* | |  | | N = 53*^1^* | |
| **Age (years)** | 42.6 (13.7)  [18.0 - 64.0] | 48.2 (15.3)  [20.0 - 81.0] | | 0.23 | | 46.1 (14.9) [18.0 - 81.0] | |
| **Sex** |  |  | | **0.033** | |  | |
| Female | 12.0 (60.0%) | 10.0 (30.3%) | |  | | 22.0 (41.5%) | |
| Male | 8.0 (40.0%) | 23.0 (69.7%) | |  | | 31.0 (58.5%) | |
| **Race** |  |  | | 0.60 | |  | |
| Caucasian | 17.0 (85.0%) | 26.0 (83.9%) | |  | | 43.0 (84.3%) | |
| African American | 2.0 (10.0%) | 3.0 (9.7%) | |  | | 5.0 (9.8%) | |
| Hispanic | 0.0 (0.0%) | 2.0 (6.5%) | |  | | 2.0 (3.9%) | |
| Asian | 0.0 (0.0%) | 0.0 (0.0%) | |  | | 0.0 (0.0%) | |
| Other | 0.0 (0.0%) | 0.0 (0.0%) | |  | | 0.0 (0.0%) | |
| Unknown | 1.0 (5.0%) | 0.0 (0.0%) | |  | | 1.0 (2.0%) | |
| **Body Mass Index (kg/m²)** | 27.5 (3.9)  [17.8 - 34.6] | 27.2 (4.4)  [16.7 - 37.8] | | 0.54 | | 27.3 (4.2)  [16.7 - 37.8] | |
| **Asthma** |  |  | | 0.14 | |  | |
| No | 5.0 (25.0%) | 15.0 (45.5%) | |  | | 20.0 (37.7%) | |
| Yes | 15.0 (75.0%) | 18.0 (54.5%) | |  | | 33.0 (62.3%) | |
| **Allergic Rhinitis** |  |  | | 0.74 | |  | |
| No | 4.0 (20.0%) | 9.0 (27.3%) | |  | | 13.0 (24.5%) | |
| Yes | 16.0 (80.0%) | 24.0 (72.7%) | |  | | 40.0 (75.5%) | |
| **Aspirin Sensitivity** |  |  | | **<0.001** | |  | |
| No | 8.0 (40.0%) | 28.0 (84.8%) | |  | | 36.0 (67.9%) | |
| Yes | 12.0 (60.0%) | 5.0 (15.2%) | |  | | 17.0 (32.1%) | |
| **Allergic Fungal Sinusitis** |  |  | | 0.090 | |  | |
| No | 15.0 (75.0%) | 31.0 (93.9%) | |  | | 46.0 (86.8%) | |
| Yes | 5.0 (25.0%) | 2.0 (6.1%) | |  | | 7.0 (13.2%) | |
| **Prior Surgery** |  |  | | 0.83 | |  | |
| No | 10.0 (50.0%) | 17.0 (53.1%) | |  | | 27.0 (51.9%) | |
| Yes | 10.0 (50.0%) | 15.0 (46.9%) | |  | | 25.0 (48.1%) | |
| **Preoperative Lund-Mackay CT Score** | 16.5 (4.6)  [9.0 - 24.0] | 17.0 (16.7)  [3.0 – 24.0] | | 0**.**18 | | 16.8 (13.4) [3.0 – 24.0] | |
| **Tissue Eosinophils per high power field** | 126.1 (64.0) [43.3 - 233.3] | 86.3 (58.5) [0.0 - 216.0] | | 0.068 | | 101.1 (63.1) [0.0 - 233.3] | |

*^1^* Mean (SD) [Range]; n (%)

*^2^* Wilcoxon rank sum test; Pearson's Chi-squared test; Fisher's exact test

CT = computed tomography

**Table S2: WikiPathways GSEA results for unadjusted differential expression analysis**

| Pathway | NES | adj p val | Genes |
| --- | --- | --- | --- |
| WP_Amplification_and_expansion_of_oncogenic_pathways_as_metastatic_traits | 1.81 | 0.01 | CXCR4CYTIP, PIK3CG, POSTN, TNC, VHL, LEF1, TCF7, TCF7L1 |
| WP_Eicosanoid_synthesis | 1.71 | 0.03 | PTGS1, ALOX5AP, PTGDS, ALOX5, TBXAS1, ALOX12, LTA4H, PNPLA3, PTGS2, PLA2G4B |
| WP_Prostaglandin_and_leukotriene_metabolism_in_senescence | 1.71 | 0.01 | PTGS1, ALOX5AP, HRAS, PTGDS, ALOX5, PLCB1, TBXAS1, ALOX12, LTA4H, SIRT1, GNAS, RB1, PTGS2, ADCY3 |
| WP_Tyrobp_causal_network_in_microglia | 1.65 | 0.00 | RGS1, CD37, GAPT, BIN2, SAMSN1, SPP1, LOXL3, RNASE6, CD84, TGFBR1, ITGB2, APBB1IP, FKBP15, DPYD, ITGAM, ADAP2, IL10RA, IGSF6, NRROS, STAT5A, GIMAP2, TMEM106A, NCKAP1L, NCF2, LYL1, HLX, PPP1R18, GAL3ST4, TYROBP |
| WP_Kisspeptinkisspeptin_receptor_system_in_the_ovary | 1.65 | 0.02 | PLCB2, PIK3CG, HRAS, RAF1, PLCB1, MMP9, PLCB4, PDK1, ARRB1, ARRB2, STAR, PRKCH, PRKCE, PIK3CD, PRKCQ, PIK3CB, PRKCB, KRAS |
| WP_microglia_pathogen_phagocytosis_pathway | 1.64 | 0.02 | CYBB, PIK3CG, PIK3R1, FCER1G, RAC2, VAV3, HCK, ITGB2, NCF4, ITGAM, C1QB, SYK, PIK3R6, NCKAP1L, NCF1, TREM1, NCF2, PIK3C3, PIK3CD, TYROBP, SIGLEC7, PIK3CB, RAC1, VAV2, C1QC |
| WP_EPO_receptor_signaling | 1.63 | 0.04 | CISH, PIK3CG, RAF1, PTPRC, JAK2, STAT5A, IRS1, PDK1, EPOR, STAT1 |
| WP_Phosphoinositides_metabolism | 1.54 | 0.03 | PLCB2, PIK3CG, PTEN, PIP4K2A, PLCB1, INPP5D, MTMR8, MTMR1, MTM1, MTMR9, PLCB4, MTMR10, PIP5K1B, PIK3R4, PIP5K1C, PIP5K1A, SBF2, MTMR11, PLCD3, PLCH2, SBF1, PIK3C3, PIK3CD, PIK3C2G, PIK3CB, MTMR4, PIP4K2B, MTMR2 |
| WP_Spinal_cord_injury | 1.50 | 0.00 | MMP12, BDNF, TNFSF13B, NOS2, GDNF, GADD45A, FOS, NR4A1, AIF1, FCGR2C, TACR1, TLR4, CDK2, PRB1, CDC42, CDK1, EGR1, CCNG1, SEMA6A, EPHA4, RGMA, AQP4, MMP9, BTG2, CDK4, SLIT3, FCGR2A, SLIT1, ZFP36, C1QB, PTPRA |
| WP_Retinoblastoma_gene_in_cancer | 1.49 | 0.02 | ANLN, RAF1, CDK2, RFC4, PLK4, DCK, CDK1, BARD1, SMC2, CCNB1, POLA1, HMGB2, POLE, CDK4, POLD3, SMC1A, HDAC1, STMN1, CDC25A, MCM7, CDC7, CCNB2, MSH6, E2F2, CDK6, SMC3, RBBP4, RFC5, HLTF, RB1, TOP2A, TTK, RBBP7, FAF1, TFDP1, CHEK1, ORC1, RRM1, RRM2, SKP2, CDT1, CDKN1B, HMGB1, POLE2, CDC45, DNMT1, MAPK13, ZNF655, KIF4A, MYC, WEE1, CCDC6, FANCG, PRKDC, CDC25B |
| WP_Cell_cycle | 1.47 | 0.01 | GADD45A, BUB3, CDK2, CDC23, CDC20, MAD2L2, CDK1, TGFB2, CCNB1, ANAPC10, SMAD4, CDK4, CDKN2D, SMC1A, CCNH, HDAC1, CDC25A, MCM7, CHEK2, CDC7, CCNB2, DBF4, ESPL1, RBX1, CDC27, E2F2, CDK6, MAD1L1, CDC25C, SMAD3, SMC3, ANAPC4, TGFB3, ATR, RB1, STAG1, CDC14B, EP300, TTK, ANAPC1, ORC5, RBL1, BUB1, TFDP1, CHEK1, PKMYT1, CCNB3, PLK1, HDAC2, ORC1, E2F5, CDKN2C, E2F4, SKP2, RBL2, FZR1, GADD45B, PTTG2, MCM5, CDKN1B |
| WP_MiRNA_regulation_of_DNA_damage_response | 1.47 | 0.04 | GADD45A, DDB2, CDK2, RAD52, CDK1, CCNG1, CCNB1, CDK4, SMC1A, CREB1, CDC25A, MCM7, CHEK2, RAD50, CCNB2, NBN, CDK6, CDC25C, APAF1, ATR, RB1, TLK1, CDC20B, BAX, CASP8, CHEK1, CCNB3, RAD9A, GADD45B, SESN1, PIDD1, CDKN1B, CYCS, CCND2, FANCD2, GADD45G |
| WP_Chromosomal_and_microsatellite_instability_in_colorectal_cancer | 1.45 | 0.05 | GADD45A, FOS, DDB2, RAC2, RAF1, TGFBR1, TGFB2, JUN, SMAD4, CSNK1A1L, MSH2, LEF1, RALB, MSH3, MSH6, BCL2, SMAD3, TCF7, TGFB3, PTGS2, TCF7L1, BAX, EXOC2, TBK1, BRAF, RALA, AXIN2, GADD45B, ARAF, RHOA, KRAS, CYCS, RAC1, GADD45G, GSK3B, TGFBR2, APPL1, MYC, RALGDS, CASP3 |
| WP_Burn_wound_healing | 1.41 | 0.04 | CXCR4, MMP3, COL1A1, TLR4, TLR9, TGFB2, TIMP2, MYD88, TNC, NOD1, DCN, TLR2, MMP9, FBN1, FLG, KRT6A, SPARC, BCL2, KDR, LY96, FGFR1, SMAD3, CCL2, FGFR3, TLR5, TGFB3, FOXE1, MMP13, FGFR2, TLR6, IL15, F13A1, KLF4, COL1A2 |
| WP_Brainderived_neurotrophic_factor_BDNF_signaling_pathway | 1.36 | 0.04 | CAMK4, NTRK2, PIK3R1, BDNF, SHC2, NTRK1, NCAM1, HRAS, NTF3, FOS, SPP1, RAF1, RASGRF1, VAV3, DOCK3, RPS6KA3, MAP2K5, CDC42, EGR1, MEF2C, JUN, KIDINS220, MTOR, CHUK, NCK1, GRIP1, TRAF6, CREB1, JAK2, STAT5A, FYN, IRS1, EIF2S2, GRIN2B, CDH2, PRKAA2, STAT1, NCF1, RANBP9, NCF2, GABRB3, RAP1A, DPYSL2, MEF2A, DLG1 |

NES = normalized enrichment score; adj p val = P values adjusted using the Benjamini Hochberg method for multiple comparisons with false discovery rate of 0.05. GSEA performed on raw expression counts unadjusted for statistically significant differences in aspirin sensitivity and biological sex.

**Table S3: WikiPathways GSEA results for adjusted differential expression analysis**

| Pathway | NES | adj p val | Genes |
| --- | --- | --- | --- |
| WP_Tyrobp_causal_network_in_microglia | 1.78 | 0.00 | RGS1, SPP1, GAPT, CD37, IL10RA, BIN2, SAMSN1, TMEM106A, NRROS, LOXL3, CD84, ADAP2, GIMAP2, ITGAM, APBB1IP, TGFBR1, DPYD, IGSF6, STAT5A, FKBP15, ITGB2, NCKAP1L |
| WP_IL2_signaling_pathway | 1.77 | 0.01 | CISH, HRAS, JUN, FOS, RAF1, PIK3R1, SYK, CBL, NMI, STAT5A, SOCS3, IL2RA, BCL2, CCND2, MYC |
| WP_Regulation_of_sister_chromatid_separation_at_the_metaphaseanaphase_transition | 1.77 | 0.04 | CDC20, ESPL1, CENPE, BUB3, BUB1, SMC3, MAD1L1, SMC1A, STAG1, BUB1B |
| WP_Interactions_between_immune_cells_and_microRNAs_in_tumor_microenvironment | 1.74 | 0.02 | IRAK4, CCL2, CD86, TGFB2, CD274, TLR4, NFKB2, TRAF6, IL2RA, PDCD1, TGFB3, IL4R, CD80, NFKB1, CCL5 |
| WP_Eicosanoid_synthesis | 1.72 | 0.04 | PTGS1, PTGDS, ALOX5AP, TBXAS1, ALOX5, PNPLA3, PLA2G4B, ALOX12, LTA4H, PTGS2 |
| WP_Hematopoietic_stem_cell_differentiation | 1.71 | 0.01 | CXCR4, CBFA2T3, IKZF1, GATA2, CD34, FOS, TRAF3IP3, TPO, FOSB, RHOH, LEF1, KITLG, ITGA2B, STAT5A, CSF1, THRB, KCNH2, NCKAP1L, IL1A |
| WP_MYD88_distinct_inputoutput_pathway | 1.71 | 0.05 | MYD88, JUN, TLR5, TLR9, TLR4, TRAF6, IL1A, TLR10, TLR2, UBE2N, NFKB1 |
| WP_Sleep_regulation | 1.69 | 0.04 | CACNA1I, PTGDS, NLGN1, FOS, PTGDR, ADORA1, STAR, GHRL, SLC29A1, HCRTR1, DRD2, PER3, DLAT |
| WP_IL5_signaling_pathway | 1.68 | 0.03 | BTK, PIK3CG, JUN, FOS, RAF1, PIK3R1, SYK, SPRED1, GSK3A, STAT5A, BCL2, JAK2, MYC, CSF2RB |
| WP_IL3_ signaling_pathway | 1.67 | 0.02 | CD69, CCR3, HRAS, JUN, FOS, RAF1, PIK3R1, CD86, SYK, HCK, CBL, INPP5D, STAT5A, IL3RA, BCL2, JAK2 |
| WP_Cell_cycle | 1.65 | 0.00 | GADD45A, CDC20, MAD2L2, CDK2, DBF4, ESPL1, CDK1, CDC25C, BUB3, CCNB1, CHEK2, E2F2, CCNB3, BUB1, ORC5, CHEK1, TGFB2, CDK6, ANAPC10, MCM7, CCNB2, RBX1, CDC23, RB1, CDC7, CDC25A, SMAD4, ORC1, CDKN2D, ORC3, SMC3, PTTG2, TTK, MCM5, CDK4, MAD1L1, CCND2, MYC, SMAD3, RBL1, TGFB3, CCNH, CCNA2, SMC1A, PCNA, STAG1, HDAC1, CCNE2, CDC27, ANAPC4, CDKN1B, FZR1, PLK1, CUL1 |
| WP_Integrated_cancer_Pathway | 1.63 | 0.02 | CDK2, BARD1, CDK1, CHEK2, CHEK1, BLM, PTEN, BAX, RB1, CDC25A, MSH2, BCL2, CDK4, MYC, RAD50, SMAD3, MSH6, CASP3, STAT1, CASP8, CDKN1B, PLK1 |
| WP_microglia_pathogen_phagocytosis_pathway | 1.62 | 0.04 | CYBB, C1QB, PIK3CG, NCF4, FCER1G, PIK3R1, PIK3R6, RAC2, SIGLEC7, ITGAM, SYK, VAV3, HCK, ITGB2, NCKAP1L |
| WP_Spinal_cord_injury | 1.61 | 0.00 | MMP12, BDNF, GADD45A, C1QB, TNFSF13B, PRB1, CDK2, GDNF, CCL2, FOS, EGR1, CCNG1, RGMA, NR4A1, CDK1, FCGR2C, NOS2, SLIT3, SEMA6A, MMP9, ZFP36, EPHA4, TLR4, TACR1, RTN4R, AIF1, RB1, LEP, BTG2, LTB, IL1A, PTGS2, CDK4, CCR2, MYC, AQP4, SLIT1, FCGR2A, CASP3, NOS1, CDC42 |
| WP_Retinoblastoma_gene_in_cancer | 1.61 | 0.02 | CDK2, BARD1, RFC4, DCK, SMC2, PLK4, ANLN, RAF1, CDK1, CCNB1, E2F2, RRM2, CHEK1, CDK6, POLA1, TOP2A, MCM7, CCNB2, RB1, CDC7, CDC25A, ORC1, STMN1, SMC3, TTK, CDK4, POLE, MYC, HMGB2, MSH6, CCNA2, HLTF, SMC1A, PCNA, RBBP4, HDAC1, FAF1 |
| WP_Synaptic_signaling_pathways_associated_with_autism_spectrum_disorder | 1.56 | 0.05 | CAMK4, NTRK2, BDNF, TSC1, HRAS, PIK3R1, PRKAB2, MTOR, PTEN, GRIN2B |
| WP_Brainderived_neurotrophic_factor_BDNF_signaling_pathway | 1.54 | 0.01 | CAMK4, NTRK2, BDNF, NCAM1, SPP1, NTF3, RASGRF1, NTRK1, HRAS, JUN, FOS, DPYSL2, EGR1, DOCK3, RAF1, KIDINS220, PIK3R1, CDH2, SHC2, MTOR, IGF2BP1, GRIN2B, NSF, VAV3, GRIP1, STAT5A, CHUK, TRAF6, CNR1, GABRB3, GRIA1, CAMK2A, MAP2K5, JAK2, NCF1, MEF2C, MEF2A, BMP2, CASP3, DLG1, IRS1, NFKB1, NCF2, MAP3K2, CDC42, STAT1, CREB1, FRS2, NCK1, NGFR |
| WP_MiRNA_regulation_of_DNA_damage_response | 1.54 | 0.05 | GADD45A, CDK2, CCNG1, CDK1, RAD52, CDC25C, DDB2, CCNB1, CHEK2, CCNB3, CHEK1, CDK6, MCM7, BAX, CCNB2, RB1, NBN, CDC25A, CDK4, CCND2, MYC, RAD50, APAF1, SMC1A, CASP3, FANCD2, CCNE2, CASP8, CREB1, CDKN1B |
| WP_Neuroinflammation_and_glutamatergic_signaling | 1.45 | 0.03 | CAMK4, BDNF, SLC38A5, LIF, IL10RA, FOS, SRR, PLCB2, TGFB2, GRIN2B, GLS, SLC2A3, GRIK2, NFKB2, TGFBR1, SMAD7, SOCS3, LRRC8C, PRKCB, SMAD4, GRIA1, PLCB1, PDHA1, IFNGR2, CAMK2A, IL1A, GRIK5, IL10RB, GLS2, BCL2, PLCB4, LRRC8A, SMAD3, TGFB3, IL4R, DLAT, SHMT1, CAMK2B, IRS1, SHMT2, SLC2A1, NFKB1, ADCY3, NOS1, PSAT1, STAT1, LRRC8D, CREB1, GRM7 |
| WP_TGFBeta_signaling_pathway | 1.44 | 0.04 | MMP12, CAV1, MAP3K7, JUN, FOS, ZEB1, RAF1, CDK1, PIK3R1, FOSB, SNW1, PARD6A, WWP1, CCNB2, MAP4K1, RBX1, DCP1A, RNF111, TGFBR1, SMAD7, SKI, TRAF6, SMAD4, COL1A2, STAMBPL1, MAP2K6, MYC, ZEB2, SMAD3, TAB1, RBL1, MEF2C, TNC, MEF2A, CREBBP, PDK1, HDAC1, HGS, CDC42, ATF3, MAP2K4, ZFYVE9, COPS5, CUL1, STRAP, BTRC, FN1, PPM1A |
| WP_Malignant_pleural_mesothelioma | 1.27 | 0.04 | MMP3, NTRK2, BDNF, WNT5A, ITGA4, FGF1, HBEGF, NTF3, ITGAV, TSC1, PRB1, CDK2, BARD1, SPARC, PIK3CG, CCL2, NTRK1, HRAS, JUN, RAF1, FGF9, CIT, ROR1, CDH19, ACTG2, PDGFB, UHRF1, CDH2, PRKAB2, AREG, MTOR, CDH11, TNNT1, WNT5B, WNT6, LEF1, MMP9, STK38L, COL4A2, PTEN, TNIK, CDH22, KITLG, EED, CD274, FLT3, PDGFC, RASSF4, RASSF2, BAX, WNT2B, GABPA, MAP4K1, CASP1, MMP14, FGF11, PIGF, CDH17, FRAT2, KIT, FGF7, CSF1, BAG2, LIN28B, ACTC1, ITGB2, COL4A3, CSF1R, CDH7, TEAD4, MAP2K5, TCF7L1, SETDB1, MAP2K6, TEAD2, BCL2, PLCB4, NLRP3, CDK4, DEPTOR, MAD1L1, CCND2, FZD10, MYC, CSNK1A1L, FOSL1, VGLL4, WNT10A, RING1, PDGFRA, FOXM1, PAK1, FOXO1, KIF23, CD44, RBBP4, PDK1, RYK, FGF17, CDH20, COL4A4, SLC2A1, NFKB1, MAP3K2, CDH3, KDR, ATF3, DDIT3, WNT4, MAP2K4, EFNA5, PRKAB1, TTI1, STAT1, CCL4, IGF2, ACTA2, CREB1, FGFR3, CSF3, ITGA6, CUL1, FGFR2, NGFR, DKK1, LAMA3, CCL5, ANGPT2, BTRC, VEGFD, FN1, PIK3CD, ANGPT4, PAK6, TELO2, NDRG1, MMP2, MDM4, AGER, CDH12, FGFR1, STK3, FGF10 |

NES = normalized enrichment score; adj p val = P values adjusted using the Benjamini Hochberg method for multiple comparisons with false discovery rate of 0.05. GSEA performed on expression counts adjusted for statistically significant differences in aspirin sensitivity and biological sex.
